# Functional Outcomes: One Year after a Cardiac Arrest

**DOI:** 10.1155/2015/283608

**Published:** 2015-09-03

**Authors:** Ketki D. Raina, Jon C. Rittenberger, Margo B. Holm, Clifton W. Callaway

**Affiliations:** ^1^Department of Occupational Therapy, School of Health and Rehabilitation Sciences, University of Pittsburgh, 5012 Forbes Tower, Pittsburgh, PA 15260, USA; ^2^Department of Emergency Medicine, School of Medicine, University of Pittsburgh, Iroquois Building, Suite 400A, 3600 Forbes Avenue, Pittsburgh, PA 15261, USA; ^3^Department of Pharmacology and Chemical Biology, School of Medicine, University of Pittsburgh, Pittsburgh, PA 15260, USA

## Abstract

*Objective*. The study aim was to characterize the time-course of recovery in impairments, activity limitations, participation restrictions, disability, and quality of life during the first year after cardiac arrest. Secondarily, the study described the associations between the instruments used to measure each of these domains. *Methods*. Measures of global disability (Cerebral Performance Category, CPC, Modified Rankin Scale, mRS), quality of life, activity limitations, participation restrictions, and affective and cognitive impairments were administered to 29 participants 1, 6, and 12 months after cardiac arrest. *Results*. Global measures of disability indicated recovery between one month and one year after cardiac arrest (mean CPC: 2.1 versus 1.69,  *P* < 0.05; mean mRS: 2.55 versus 1.83, *P* < 0.05). While global measures of disability were moderately associated with participation, they were poorly associated with other measures. The cohort endorsed depressive symptomatology throughout the year but did not have detectable cognitive impairment. *Conclusions*. Recovery from cardiac arrest is multifaceted and recovery continues for months depending upon the measures being used. Measures of global disability, reintegration into the community, and quality of life yield different information. Future clinical trials should include a combination of measures to yield the most complete representation of recovery after cardiac arrest.

## 1. Introduction

Cardiac arrest (CA) is the sudden, unexpected, cessation of cardiac function and is confirmed by the absence of pulse and breathing [1]. Worldwide, the average incidence rate for CA is 55 per 100,000 persons with survival rates as high as 22% in some subgroups [2, 3]. Aggressive treatment has been associated with improved survival after CA [1, 4–8]. Short and long-term impairment (dysfunction in physiological functions and anatomical parts of the body) and disability (difficulties experienced in the execution of everyday activities and involvement in life situations) are critical patient-centered outcomes. A recent consensus statement from the American Heart Association emphasizes the need to include multiple impairment and disability measures with longer-term endpoints to characterize recovery among cardiac arrest survivors [9]. A similar need has been echoed by the Society of Critical Care Medicine at a stakeholder's conference for improving long-term outcomes after discharge from intensive care unit [10].

Individuals resuscitated from CA sometimes have significant cognitive impairments that may lead to disability [11]. Many surviving patients report decreased capacity to perform everyday activities, fail to return to work, and experience fair to poor quality of life (QOL) [12–16]. While prior studies are limited by the use of short follow-up times after CA or focus on only one or two measures to assess recovery in terms of the individuals disability and quality of life [14, 16], recent data suggest that recovery continues for months after CA. A recent study by Larsson et al. (2014) reported that while QOL continues to improve up to 6 months after CA, survivors continue to report low physical and mental quality of life compared to the general population [17].

The aim of the study was to characterize the time-course of recovery in function, disability, and participation 1 year after CA. This study is unique in that we prospectively evaluated a cohort of patients for one year after CA using multiple measures of affective and cognitive impairments, global disability, QOL, activity limitations, and participation restrictions. We tested the hypotheses that impairments, global disability, QOL, activity limitations, and participation restrictions improved over time after CA. Secondarily, we described the associations between the instruments used to measure each of these domains.

## 2. Materials and Methods

### 2.1. Design

In this longitudinal observational study, data were collected at discharge from hospital and 1, 6, and 12 months after the CA. The 12-month assessment was considered the main endpoint for the study. CA was defined as a loss of pulse requiring chest compressions by a professional responder, rescue shock, or both.

### 2.2. Participants

Adults (18 years and older) who had survived CA for more than 3 days and were admitted to a tertiary care facility were recruited for this study. Subjects who sustained an in- or out-of-hospital CA were included in the study. Arrests occurring in the emergency department were considered in-hospital CA. Subjects sustaining a CA due to trauma or following cerebrovascular accident were excluded from the study because the pathophysiology, epidemiology, and expected outcomes from traumatic CA differ from medical CA. Informed consent was obtained from subjects or their authorized representatives. The study was approved by the Institutional Review Boards at the University of Pittsburgh and at Mercy Hospital of Pittsburgh, Pittsburgh, Pennsylvania, United States.

### 2.3. Instrumentation

Longitudinal data were obtained at various time points using multiple instruments (see Supplementary Material available online at http://dx.doi.org/10.1155/2015/283608).

Global disability was measured using the Cerebral Performance Category (CPC) [18, 19], Modified Rankin Scale (mRS) [20, 21], and the Extended Glasgow Outcome Scale (GOSE) [22, 23]. A structured interview format was used to obtain data for the mRS and GOSE [21, 23]. The CPC is a 5-category scale that measures neurological recovery after CA. A score of 1 indicates consciousness and good cerebral performance while a score of 5 indicates death. The interrater reliability for the CPC scores is good (*k* = 0.87) [24]. The MRS is a 7-point scale that has been used to measure disability after stroke and traumatic brain injury [20]. It has faced similarity with the CPC. A score of 0 indicates no symptoms at all and a score of 6 indicates death. The interrater and intrarater reliability for the structured interview used in the study were *k*_*w*_ = 0.91 and *k*_*w*_ = 0.95–0.99, respectively [20]. The GOSE is an 8-point scale that has been used to assess outcomes after acute brain injury. A score of 8 indicates upper good recovery while a score of 0 indicates death. Interrater reliability for the structured interview was *k*_*w*_ = 0.85 [21]. QOL was measured using the Health Utilities Index, Mark 3 (HUI-3) [25, 26]. The HUI-3 links QOL to disability by assessing 8 constructs (e.g., vision, mobility, dexterity, and emotion). It is a 41-item interviewer-administered questionnaire that yields scores ranging from 1 (perfect health) to 0 (death). Scores less than 0 describe a health state worse than death. Additionally, scores from 0.99 to 0.89 represent mild disability, scores from 0.88 to 0.70 represent moderate disability, and scores <0.70 represent severe disability (Furlong & Feeney, Personal Communication, January 3, 2008). Internal consistency for the HUI-3 is good (*α* = 0.88) [25]. Three-month test-retest reliability for the tool was found to be acceptable (ICC = 0.75) [25].

Depressive symptoms were measured using the 30-item Geriatric Depression Scale (GDS) [27]. Scores on the GDS range from 0 to 30, with 0 signifying no depression and 30 signifying severe depression. Scores ranging from 1 to 10 are considered normal, while scores greater than 11 are indicative of depressive symptomatology [27]. Internal consistency for the GDS is good (*α* = 0.94) and test-retest reliability has been excellent over two weeks (ICC = 0.98) [27]. The Adult Lifestyle and Function Interview-Mini Mental State Examination (ALFI-MMSE) and Telephone Interview of Cognitive Status (TICS) were used as measures of cognitive impairment [28, 29]. Both instruments were selected because they could be administered over the telephone. Scores for the 12-item ALFI-MMSE range from 0 to 22 with 0 indicating severe impairment and 22 indicating no cognitive impairment. A cutoff score of 17 indicates cognitive impairment [29]. The ALFI-MMSE is strongly correlated with the MMSE (*r* = 0.85; *P* < 0.001) [30]. The TICS consists of 11 items. Scores for the TICS range from 0 to 41 with 0 indicating severe cognitive impairment and 41 indicating no cognitive impairment. A cutoff score of 28 is indicative of cognitive impairment [29]. One-month test-retest reliability for the TICS has been high (*r* = 0.90, *P* < 0.001) [29].

Activity limitations were measured using the interview version of the Performance Assessment of Self-Care Skills (PASS) [31, 32]. The PASS consists of 26 basic and instrumental activities of daily living requisite for independent living in the community. The PASS yields scores for habit (“does patient perform the activity routinely?”) and skill (“can the patient perform the activity?”). Each score ranges from 0 to 3, with 0 indicating total dependence and 3 indicating independence. Test-retest reliability for the PASS is high (*r* = 0.82, *P* < 0.05) [32].

Participation restrictions were measured using the Reintegration to Normal Living Index (RNLI) [33]. Scores on the 11-item RNLI range from 0 to 22. A score of 0 indicates poor reintegration while a score of 22 indicates reintegration into the community. Internal consistency for the RNLI is good (*α* = 0.92) [33].

### 2.4. Procedures

Review of medical charts at the time of discharge from the hospital determined the chart-review CPC (c-CPC) and mRS (c-mRS) scores. Physicians (Jon C. Rittenberger, Clifton W. Callaway) who are experienced in the examination of post-CA patients and did not have in-person contact with the participants after they were discharged from the hospital used written instruments to determine c-CPC and c-mRS scores. When specific data about these activities could not be found or when notes were conflicting, the raters assumed the worst outcome.

The 1-, 6-, and 12-month follow-ups were conducted via an in-person or telephone interview with the patient by an occupational therapist (Ketki D. Raina), with prior experience in the administration of each of these instruments. If the patient was unable to communicate, a proxy was interviewed. Interrater agreement between patient and proxies was acceptable for mRS, GOSE, and HUI-3 [17, 18, 21]. The GDS, ALFI-MMSE, and TICS were not administered to the proxy.

### 2.5. Statistics

Descriptive statistics were generated for all measures. Demographic, injury severity, c-CPC, and c-mRS scores from participants who did and did not complete the study were compared using parametric (*t*-tests) and nonparametric (Chi-square) tests as appropriate. To compare changes in measures over time, one-way repeated-measures Analysis of Variance (ANOVA) was conducted. If analysis revealed a violation of Mauchly's sphericity assumption, adjustments were made to the ANOVA results, using the Greenhouse-Geisser epsilon. Post hoc analyses were also conducted using dependent samples *t*-tests. Effect sizes were expressed using Cohen's *d*. Effect size values ranged from small (*d* = 0.20) to medium (*d* = 0.50) to large (*d* = 0.80) [34]. Spearman rho correlation coefficients were calculated to examine the associations between the multiple measures at the 12-month time point. Correlations ranging from 0.00 to 0.25 indicated a poor relationship; those from 0.26 to 0.50 indicated a fair relationship; values of 0.51 to 0.75 indicated a moderate relationship; and values above 0.76 indicated a good relationship [35]. Statistical calculations were performed using PASW v18 statistical software (PASW 18.0, IBM Corporation, Somers, NY, USA).

## 3. Results

### 3.1. Patient Population

Forty-nine subjects were entered in the study. The total first-year mortality after CA was 5 persons (10%). Mortality at the 1st, 6th, and 12th month was 4 (8%), 1 (2%), and 0, respectively. Four subjects (8%) refused to continue participation in the study and 11 subjects (22%) could not be contacted because they either had moved away after the CA or may have moved to nursing facility without any forwarding information. Analysis was restricted to data for the remaining 29 participants over one year. There were no differences in demographic features, arrest characteristics, and c-CPC and c-mRS scores between participants and nonparticipants in the study (Table 1).

Table 1 includes demographic and CA data for 29 participants. Mean age for the sample was 60.8 ± 16.3 years. Participants were more likely to be Caucasian males and to have sustained a witnessed, out-of-hospital, ventricular fibrillation CA. Thirteen participants received therapeutic hypothermia. Participants spent a median of 11 days (IQR: 8.8–18.0) in the hospital, of which 4 (IQR: 3.0–7.0) were in the ICU. Prior to the CA, all participants were independent in activities of daily living (e.g., toileting, walking), 86% were independent in instrumental activities of daily living (e.g., shopping, meal preparation, and travel), 55% worked or were seeking work, and 100% participated in social and leisure activities.

### 3.2. Longitudinal Data

Longitudinal data for all measures are presented in Table 2. Mean scores for CPC and mRS at the four time points met the definition for good outcomes (1-2 for the CPC, 0–3 for the mRS). Mean scores for GOSE at 1, 6, and 12 months indicated lower moderate disability, while mean scores for the HUI-3 indicated severe disability. The participants demonstrated no cognitive impairment (ALFI-MMSE and TICS), but they endorsed depressive symptoms at the 1-, 6-, and 12-month time points. The PASS Habit and Skill scores indicated that participants had difficulty in performing more than one daily activity or needed assistance from another individual in performing at least one daily activity. Finally, the RNLI scores indicated that participants perceived that they were not fully integrated into the community.

**Figure 1:**
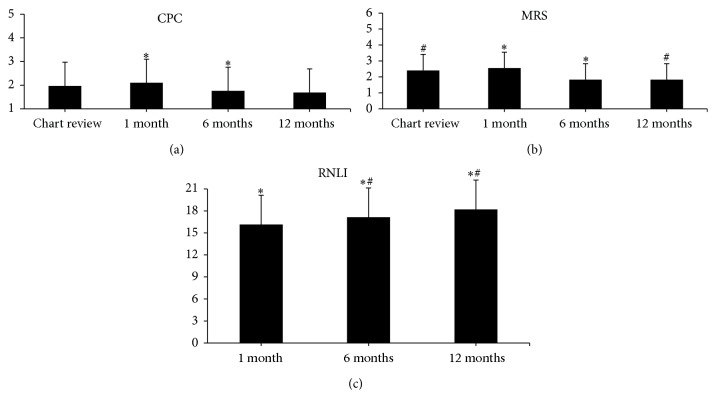
Bar graphs for longitudinal data. (a) demonstrates the longitudinal CPC data. (b) illustrates the longitudinal mRS data. (c) illustrates the longitudinal RNLI data. CPC: Cerebral Performance Category. mRS: Modified Rankin Scale, CPC: Cerebral Performance Category, and RNLI: Reintegration to Normal Living Index. ^*∗*^ *P* < 0.05 between time points; ^#^ *P* < 0.05 between time points.

There were significant main effects of time on CPC, mRS, and RNLI (Table 2). Post hoc paired samples *t*-tests showed that CPC scores were significantly better at 6 months compared to 1 month (Figure 1(a)). Similarly, mRS scores were significantly better at 6 and 12 months compared to 1 month and chart review, respectively (Figure 1(b)). A post hoc analysis of RNLI scores revealed significantly better scores at 12 months compared to 1 and 6 months (Figure 1(c)).

An examination of the effect sizes (Table 3) revealed medium effect sizes for differences between 1 and 6 months for HUI-3, PASS-SR Skill, and RNLI scores; between 6 and 12 months for PASS-SR Skill and RNLI scores; and between 1 and 12 months for PASS-SR Habit scores. Large effect sizes were found for differences between 1 and 6 months for CPC and mRS scores and between 1 and 12 months for CPC, mRS, PASS-SR Skill, and RNLI scores. The effect sizes for all other comparisons were small.

### 3.3. Associations among Measures

Relationships (Table 4) among the global measures of disability ranged from moderate (GOSE and CPC, GOSE and mRS) to good (mRS and CPC). Similarly, the strength of the relationships among RNLI and global disability measures was moderate (RNLI and CPC, RNLI and mRS) to good (RNLI and GOSE). Additionally, moderate relationships were seen between HUI-3 and the PASS-SR Skill, HUI-3 and RNLI, and PASS-SR Skill and the RNLI. The strength of relationships for all other comparisons was fair to poor.

## 4. Discussion

In this study, we describe the time-course of recovery using multiple measures in individuals who had survived CA. We also describe the relationship among the measures. Our analyses revealed that scores on three measures improved over time: CPC, mRS, and RNLI. Other measures indicated persistent disability and depressed mood after CA.

The results indicate that recovery continues for months after hospital discharge, but they indicate that many subjects do experience long-term disability. Mean CPC and mRS scores improved more between 1 and 6 months (*d* = 0.73) than between 6 and 12 months (*d* = 0.21). This suggests that most recovery in terms of disability occurs within the first 6 months after CA. For example, the mean values for both CPC and mRS were greater than 1 at 12 months. While GOSE scores did not vary over the year, the mean GOSE score at 12 months was 5.83. A score of 5 on the GOSE indicates lower-moderate recovery. These findings are similar to those recently reported by Larsson et al., in a cohort of survivors who had received therapeutic hypothermia [17]. At hospital discharge, 73% of survivors reported that their overall health status was lower than the Swedish general populations, while at 6 months after cardiac arrest, 41% patients continued to report that their overall health status was lower than the Swedish general population. Despite this poor recovery, rehabilitation after cardiac arrest has received little attention in the literature. A search of the literature revealed 2 articles that have focused on neurological recovery for individuals who have severe neurological disability after a cardiac arrest [15, 36]. However, our data show that a proportion of CA survivors do experience moderate disability and suggest a need for rehabilitation services to target this population.

The strength of the relationships among the global measures of disability (CPC, mRS, and GOSE) was moderate to good suggesting that they measure a similar construct. The GOSE may be preferred to the CPC and mRS because it has a better-defined 8-point scale. However, the mRS was able to detect changes over time, which GOSE did not capture. Both the GOSE and the mRS also take into consideration prior disability, which makes these measures preferable to CPC for measuring the effect of the CA.

The persistent disability after CA may influence participation in daily activities, as reflected by improving scores on RNLI and PASS over time and the associations of these measures with CPC, mRS, and GOSE. Individuals with functional impairments may have decreased work capacity, be unable to return to work, have reduced participation in social and leisure activities, and have poor family relationships. Other studies reported a rate of return to work as low as 13% after a CA [12, 13, 37]. A reduction in work capacity has a direct influence on not only the individual but also the family finances and dynamics. Early consultation with social services may be indicated if an individual is at risk for not returning to work. Additionally, CA survivors also complain of more social problems that they attribute to an alteration in their routine [38].

While the global measures reported mild to moderate disability, HUI-3 scores at 1, 6, and 12 months indicated severe decrease in health utility. Even though a moderate effect size (*d* = 0.43) indicated that individuals perceived less disability at 6 months compared to 1 month, scores were still in the severe range. One explanation for the contrasting results between measures of perceived health and global disability may be the different information that each of these measures collects. This was represented by the poor to fair relationships between the HUI-3 and the global disability measures. The HUI-3 consists of 41 questions that query the individual about various impairments, such as vision, hearing, dexterity, affect, cognition, and overall health. The global measures in contrast, mainly, assess the individuals' perceptions about their ability to perform activities in the home and community, work, social and leisure, and residual impairments. Additionally, global disability measures, such as the CPC, also contain poorly defined, subjective criteria. Previous research has shown poor concurrent validity between the CPC and the HUI-3, wherein each criterion on the CPC encompasses a wide range of HUI-3 scores [24]. Compared to the CPC, the structured interview format for the mRS and GOSE is likely to yield a more accurate characterization of the individual's disability [21, 23]. Future clinical trials may need to include both global disability measures, such as mRS and GOSE, as well as a health-related QOL measure, such as HUI-3, because they capture and report different types of information. We note that prior literature using SF-36 did not show impairments [39–41]. SF-36 is an overall quality of life measure, while HUI-3 focuses on health-related quality of life. The additional domains assessed by the HUI-3 may have permitted detection of impairments that are not assessed by SF-36.

Scores on RNLI seemed to be more reflective of the global disability measures (*r* ranged from −0.69 to 0.77), as CA survivors' perceived decreased disability and increased reintegration into the community throughout the year. The RNLI captures information on individuals' abilities to move around within their environment, meet self-care needs, and return to work and social activities. Hence, while individuals were still impaired at 12 months (HUI-3 scores), they were more likely to have adapted to their impairments and participate in everyday life.

While cognitive impairment is one of the most common sequelae of CA, surprisingly, our sample did not exhibit any cognitive impairments on the scales we used [11, 42]. A systematic review has reported that CA survivors are most likely to have deficits in memory, attention, and executive function [11]. We selected the MMSE and the TICS because they could be administered over the telephone and they have been used previously with CA survivors. However, prior studies have used sophisticated neuropsychological batteries to detect cognitive impairment in CA survivors [11]. Very large deficits would be required to produce changes in MMSE and TICS, and performance on these measures cannot exclude the presence of more subtle cognitive impairments.

Of particular concern is that individuals endorsed depressive symptomatology throughout the course of one year. CA survivors are known to endorse signs of anxiety and depression that persist beyond 6 months and 36 months after CA [5, 11, 43]. Depressed mood increases the risk for future cardiac events, is strongly associated with disability, and leads to poor rehabilitation outcomes [44, 45]. Hence, healthcare professionals working with CA survivors may need to aggressively assess anxiety and depression and refer patients to mental health practitioners if depression is suspected. While our study used the Geriatric Depression Scale to assess depressive symptomatology, other studies have also used the Center for Epidemiological Studies Depression Scale to measure depression and State-Trait Anxiety Inventory (STAI) to measure anxiety in cardiac arrest survivors [11].

In summary, our findings indicate that various measurements change over time. Given these dynamic changes during the first year, post-CA patients may benefit from serial examinations at specific time epochs, permitting adaptations in rehabilitation strategies based on the results at these time points. For example, our findings indicate an improvement in the global disability over the first 6 months after the CA, but it appears that the rehabilitation strategy may need to be modified after 6 months to promote reintegration into society.

The findings of this study may be limited by its sample size, selection bias, selection of outcome measures, and a 1-year follow-up period. While our study has a small sample size (*n* = 29), we collected very high-resolution data using multiple measures at various time points. Hence, the results of our study provide a characterization of functioning and disability one year after a CA. The subjects who completed participation in our study may be less ill than in other cohorts, because the mortality rate in our study was low compared to the 20–30% postdischarge 1-year mortality rates reported in other studies [13, 37, 46, 47]. Part of the selection for less ill subjects may have resulted from the fact that we enrolled subjects only after they had survived for at least 72 hours and for whom the patient or proxy was willing to consent to one-year follow-up. Prior literature has demonstrated that failure to awaken from coma is the most common cause for the withdrawal of care in patients dying during the first 72 hours following resuscitation from cardiac arrest [48]. Hence, individuals who were more neurologically devastated may not have enrolled in the study. We did not detect any difference in the baseline characteristics of participants and subjects who withdrew or who were lost to follow-up. It is possible that the persistent disability observed in our sample may actually be worse for many other CA survivors. The outcome measures used in this study are not commonly obtained while in hospital and their inclusion in everyday practice may be perceived as burdensome. This begs for the development of a screening tool to quickly and accurately assess critical care patients prior to their discharge from hospital and over the long term. Lastly, we sought to characterize the time-course of recovery 1-year after the CA. A longer follow-up time period of up to 5 years may have provided us with greater insight into recovery and residual deficits in this population.

## 5. Conclusion

Recovery after CA is characterized by functional impairments, disability, and participation, each of which is measured by different instruments. Recovery continues for months after CA. Despite recovery, depressive symptoms are common even 1 year after CA. Future clinical trials should include a combination of measures, perhaps including refined measures of depression and cognitive impairment, to yield a complete representation of recovery after CA.

## Supplementary Material

The Table provides a listing of the measures administered at Chart Review, 1 month, 6 months, and 12 months post- cardiac arrest.

## Figures and Tables

**Table 1:** Demographic data for participants, nonparticipants (who withdrew from study or were lost to follow-up), and nonsurvivors.

	Participants (*n* = 29)	Nonparticipants (*n* = 15)	Nonsurvivors (*n* = 5)
Age in years (SD)	60.8 (16.3)	56.2 (13.4)	59.6 (22.4)
Male gender, *n* (%)	18 (62)	10 (67)	4 (80)
Race, *n* (%)			
Caucasian	27 (93)	13 (87)	5 (100)
African American	2 (7)	2 (13)	0 (0)
OHCA, *n* (%)	17 (59)	12 (85)	3 (60)
Presumed cardiac etiology, *n* (%)	25 (86)	14 (93)	4 (80)
Witnessed, *n* (%)	25 (86)	11 (73)	5 (100)
Bystander CPR, *n* (%)	25 (88)	5 (56)	3 (60)
Rhythm, *n* (%)			
VF/VT	22 (76)	10 (71)	3 (60)
PEA	4 (14)	3 (21)	1 (20)
Asystole	2 (7)	0 (0)	0 (0)
Unknown	1 (3)	1 (7)	1 (20)
Rescue shocks delivered (SD)	1.54 (1.37)	1.91 (1.30)	3.20 (2.86)
Hypothermia, *n* (%)	13 (45)	9 (60)	1 (20)
Median duration of coma in days (IQR)	1.0 (0.8–1.80)	1.5 (1.0–2.4)	1.6 (0.0–12.6)
Median duration of intubation in days (IQR)	2.0 (1.0–3.5)	3.5 (1.0–5.0)	6.0 (1.3–12.3)
Median ICU length of stay in days (IQR)	4.0 (3.0–7.0)	6.5 (4.0–13.0)	6.0 (2.0–17.5)
Median total length of stay in days (IQR)	11.0 (8.8–18.0)	11.0 (8.0–25.5)	15.0 (10.0–30.5)

SD = standard deviation; OHCA = out-of-hospital cardiac arrest; CPR = cardiopulmonary resuscitation; VF/VT = ventricular fibrillation/ventricular tachycardia; PEA = pulseless electrical activity; IQR = interquartile range.

**Table 2:** Descriptive and one-way repeated-measures ANOVA for multiple measures.

Measure (score range)	Chart review	1 month	6 months	12 months	*F* (df)
CPC (1–5)^a^	1.97 (0.94)	2.10 (1.01)	1.76 (0.87)	1.69 (0.81)	3.93^*∗*^ (2.4)
mRS (0–6)^a^	2.41 (1.40)	2.55 (1.53)	1.83 (1.49)	1.83 (1.39)	6.17^*∗*^ (2.4)
GOSE (0–8)^b^		5.52 (3.52)	5.66 (2.00)	5.83 (2.00)	0.12 (1.2)
HUI-3 (0-1)^b^		0.59 (0.42)	0.68 (0.35)	0.66 (0.35)	1.50 (1.46)
GDS (0–30)^a^		11.08 (4.07)	12.18 (3.62)	11.86 (3.85)	0.79 (1.3)
ALFI-MMSE (0–22)^b^		20.75 (2.03)	20.82 (2.51)	20.96 (2.03)	0.37 (2.0)
TICS (0–41)^a^		35.43 (5.59)	36.00 (4.46)	35.57 (5.39)	0.44 (1.6)
PASS-SR Habit (0–3)^b^		2.17 (0.86)	2.26 (0.88)	2.30 (0.80)	1.66 (1.6)
PASS-SR Skill (0–3)^b^		2.53 (0.75)	2.65 (0.73)	2.70 (0.73)	0.80 (1.0)
RNLI (0–21)^b^		16.14 (6.22)	17.14 (6.10)	18.21 (5.51)	5.39^*∗*^ (2.0)

Note: CPC = Cerebral Performance Category; mRS = Modified Rankin Scale; HUI-3 = Health Utilities Index, Mark 3; GDS = Geriatric Depression Scale; ALFI-MMSE = Adult Lifestyle and Function Interview-Mini Mental State Examination; TICS = Telephone Interview of Cognitive Status; GOSE = Glasgow Outcome Scale Extended; RNLI = Reintegration to Normal Living Index; PASS-SR Habit = Performance Assessment of Self-Care Skills Self-Report Habit; PASS-SR Skill = Performance Assessment of Self-Care Skills Self-Report Skill.

a: higher scores indicate greater impairment/disability. b: lower scores indicate greater impairment/disability.

^*∗*^ *P* < 0.05.

**Table 3:** Effect size (Cohen's *d*) over time for multiple measures.

Measure (score range)	Chart review, 1 month	1 month–6 months	6 months–12 months	1 month–12 months
CPC (1–5)^a^	0.21	0.73	0.21	0.86
mRS (0–6)^a^	0.17	0.84	0.00	0.86
GOSE (0–8)^b^	201	0.05	0.18	0.11
HUI-3 (0-1)^b^	—	0.43	0.19	0.26
GDS (0–30)^a^	—	0.29	0.19	0.19
ALFI-MMSE (0–22)^b^	—	0.09	0.13	0.24
TICS (0–41)^a^	—	0.23	0.25	0.05
PASS-SR Habit (0–3)^b^	—	0.28	0.15	0.54
PASS-SR Skill (0–3)^b^	—	0.62	0.47	0.76
RNLI (0–21)^b^	—	0.39	0.59	0.79

Note: CPC = Cerebral Performance Category; mRS = Modified Rankin Scale; HUI-3 = Health Utilities Index, Mark 3; GDS = Geriatric Depression Scale; ALFI-MMSE = Adult Lifestyle and Function Interview-Mini Mental State Examination; TICS = Telephone Interview of Cognitive Status; GOSE = Glasgow Outcome Scale Extended; RNLI = Reintegration to Normal Living Index; PASS-SR Habit = Performance Assessment of Self-Care Skills Self-report Habit; PASS-SR Skill = Performance Assessment of Self-Care Skills Self-Report Skill.

a: higher scores indicate greater impairment/disability. b: lower scores indicate greater impairment/disability.

**Table 4:** Spearman rho correlation coefficients between measures at 12 months.

	CPC	mRS	GOSE	HUI	GDS	MMSE	TICS	PASS-H	PASS-S	RNLI
CPC	1.00									
mRS	0.85^*∗*^	1.00								
GOSE	−0.67^*∗*^	−0.68^*∗*^	1.00							
HUI	−0.35	−0.48^*∗*^	0.45^*∗*^	1.00						
GDS	0.29	0.38^*∗*^	−0.34	−0.45^*∗*^	1.00					
MMSE	−0.16	−0.10	0.25	0.23	−0.19	1.00				
TICS	−0.14	−0.24	0.45^*∗*^	0.37	−0.27	0.46^*∗*^	1.00			
PASS-H	−0.49^*∗*^	−0.39^*∗*^	0.19	0.40^*∗*^	−0.37	−0.02	0.04	1.00		
PASS-S	−0.50^*∗*^	−0.46^*∗*^	0.43^*∗*^	0.66^*∗*^	−0.32	0.01	0.09	0.50^*∗*^	1.00	
RNLI	−0.71^*∗*^	−0.69^*∗*^	0.77^*∗*^	0.54^*∗*^	−0.39^*∗*^	0.18	0.29	0.42^*∗*^	0.59^*∗*^	1.00

NOTE: CPC = Cerebral Performance Category; mRS = Modified Rankin Scale; HUI = Health Utilities Index, Mark 3; GDS = Geriatric Depression Scale; ALFI-MMSE = Adult Lifestyle and Function Interview-Mini Mental State Examination; TICS = Telephone Interview of Cognitive Status; GOSE = Glasgow Outcome Scale Extended; RNLI = Reintegration to Normal Living Index; PASS-H = Performance Assessment of Self-Care Skills Self-Report Habit; PASS-S = Performance Assessment of Self-Care Skills Self-Report Skill.

^*∗*^ *P* < 0.05.
