# Supplementary material for: Functional Outcomes: One Year after a Cardiac Arrest
Source: Biomed Res Int. 2015 Sep 3;2015:283608. doi: 10.1155/2015/283608 (PMC4573239; doi:10.1155/2015/283608)
Supplement: Supplementary file 1 — The Table provides a listing of the measures administered at Chart Review, 1 month, 6 months, and 12 months post- cardiac arrest. [file 283608.f1.doc]

Table. Timing of Measures

| Measure | Chart Review | 1 month | 6 months | 12 months |
| --- | --- | --- | --- | --- |
|  |  |  |  |  |
| CPC | X | X | X | X |
| mRS | X | X | X | X |
| GOSE |  | X | X | X |
| HUI-3 |  | X | X | X |
| GDS |  | X | X | X |
| ALFI-MMSE |  | X | X | X |
| TICS |  | X | X | X |
| PASS-SR Habit |  | X | X | X |
| PASS-SR Skill |  | X | X | X |
| RNLI |  | X | X | X |

Note: CPC = Cerebral Performance Category; mRS = Modified Rankin Scale; HUI-3 = Health Utilities Index, Mark 3; GDS = Geriatric Depression Scale; ALFI-MMSE = Adult Lifestyle and Function Interview – Mini Mental State Examination; TICS = Telephone Interview of Cognitive Status; GOSE = Glasgow Outcome Scale Extended; RNLI = Reintegration to Normal Living Index; PASS-SR Habit = Performance Assessment of Self-Care Skills Self-report Habit; PASS-SR Skill = Performance Assessment of Self-Care Skills Self-report Skill.
